# An International Pilot Study of Self-Reported Quality of Life in Outpatient and Inpatient Mental Health Settings

**DOI:** 10.3389/fpsyt.2021.719994

**Published:** 2021-08-05

**Authors:** Johanna de Almeida Mello, Hao Luo, Alice Hirdes, Jyrki Heikkilä, Benoite Umubyeyi, Darius Gishoma, Margaret Saari, John P. Hirdes, Chantal Van Audenhove

**Affiliations:** ^1^LUCAS Center for Care Research and Consultancy, KU Leuven University, Leuven, Belgium; ^2^Department of Social Work and Social Administration, The University of Hong Kong, Hong Kong, China; ^3^Graduate Program in Health Promotion, Human Development and Society, Lutheran University of Brazil, Canoas, Brazil; ^4^Division of Psychiatry, Turku University Hospital, Turku, Finland; ^5^College of Medicine and Health Sciences, University of Rwanda, Kigali, Rwanda; ^6^SE Research Center, SE Health, Markham, ON, Canada; ^7^Lawrence S. Bloomberg Faculty of Nursing, University of Toronto, Toronto, ON, Canada; ^8^School of Public Health and Health Systems, University of Waterloo, Waterloo, ON, Canada; ^9^Academic Center for General Practice in the Department of Public Health and Primary Care, KU Leuven University, Leuven, Belgium

**Keywords:** quality of life, benchmarking, mental health services, patient reported experience measures, international comparisons

## Abstract

**Introduction:** Measuring quality of life (QoL) is essential to understand how clients perceive their care. In practice, many instruments are in place to identify mental health diagnoses and measure treatment outcomes, but there are fewer standardized instruments to routinely collect information about self-reported QoL, especially across different mental health settings. Moreover, existing tools have been criticized for being built from the perspective of care professionals rather than the users' perspective. The 23-item Self-Reported interRAI-QoL Survey for Mental Health and Addictions (interRAI SQoL-MHA) tackles these issues, as it is based on self-reported measures and has proven validity across settings and countries.

**Objective:** The aim of this study is to assess and compare QoL across settings and explore associations between dimensions of self-reported QoL and some items from the interRAI SQoL-MHA in a multinational sample.

**Settings:** Inpatient and community mental health services.

**Methods:** Data were collected from organizations in Belgium, Finland, Russia, Brazil, Rwanda, Canada and Hong Kong. Logistic regression models were constructed using each domain scale of the interRAI SQoL-MHA (relationship, support, hope, activities and relationship with staff) as dependent variables.

**Results:** A total of 2,474 people (51.2% female, 56.7% of age 45 or older) were included in the study. A benchmark analysis showed the samples that performed above the benchmark line or below. The models yielded significant odds ratios among the domain scales, as well as for the items of the interRAI SQoL-MHA, with positive associations for the items “work and education opportunities” and “satisfied with services”, and inverse associations for the items “financial difficulties” and for the inpatient setting.

**Conclusion:** The analysis of associations between the determinants offers relevant information to improve mental health care and clients' perceived quality of life. Information about the determinants can help policymakers to design interventions to improve care outcomes, as well as provide more possibilities for integration into the community. The interRAI SQoL-MHA is innovative, as it can be linked to the third generation interRAI MH and Community MH-instruments, to be used in different mental health care settings, combining the objective and subjective QoL domains.

## Introduction

Over the past two decades, a shift has taken place in the approach to mental health care, moving from an emphasis on the reduction of symptoms, based on pathology and illness, to a more comprehensive and holistic approach (1, 2). The definition put forward by Anthony (1993) was a key milestone for this shift, where recovery was described as “*a deeply personal, unique process of changing one's attitudes, values, feelings, goals, skills, and/or roles. It is a way of living a satisfying, hopeful, and contributing life even with limitations caused by illness. Recovery involves the development of new meaning and purpose in one's life as one grows beyond the catastrophic effects of mental illness* (3).” This new vision brought the client's perspective into the foreground and was a reaction against the singular clinical vision of care professionals, where patients and former patients felt that important aspects were missing in the delivered care (4, 5). Since then, personal well-being, recovery, social functioning and quality of life (QoL) have become essential elements in mental health rehabilitation (6–11). In scientific literature, studies state that evaluating mental health rehabilitative interventions means primarily to determine whether these interventions have the potential to increase users' quality of life (12). In a broader vision, interventions should improve users' sense of well-being, health status as well as satisfaction with life circumstances, including access to resources and opportunities (13, 14). According to Thornicroft and Slade (15), it is the point of view of the service users that counts most in deciding which outcomes should be assessed when evaluating mental health interventions. They agree that quality of life is not closely related to users' needs as rated by the staff, but is closely associated with unmet needs as rated by service users (16, 17). This highlights the importance of users' self-rated measures of QoL.

This expansion of focus is reflected in the Institute for Healthcare Improvements Triple Aim initiative that emphasizes the need for approaches to health service delivery that improve the patient experience of care, improve health of populations, and reduce costs of health care simultaneously (18). The opportunity to engage in international benchmarking on the quality of life of service recipients depends on the availability of standardized measures that are cross-nationally applicable. International comparisons can provide evidence of what is possible in settings with differing resources, and they can provide natural policy experiments to evaluate alternative approaches to service provision (19).

In practice, many instruments are in place to identify mental health diagnoses and measure treatment outcomes, but there fewer standardized instruments routinely collect information about self-reported QoL, especially across different types of mental health settings (20). Existing tools to measure QoL have also been criticized for taking the perspective of care professionals rather than the users' perspective (21, 22). The 46-item interRAI Self-Reported Quality of Life Survey for Mental Health and Addictions (interRAI SQoL-MHA) (23) tackles these aspects, as it is a self-report instrument that can be applied to different types of organizations delivering inpatient care or care in the community. This instrument has a psychosocial perspective of QoL based on the individual's sense of well-being, containing a total of four domain scales: “relationship”, “hope”, “support”, and “activities”, with an additional 8-item “relationship with staff” scale. The tool can be best applied in conjunction with the interRAI Mental Health and Community Mental Health care instruments to include both the subjective and objective perspectives of a person's QoL (24).

While several factors have been associated with subjective QoL, lower capacity for everyday functioning and having a greater severity of depressive and anxiety symptoms have been associated with poor subjective QoL (25, 26). However, literature shows that symptom reductions alone usually do not result in significant improvements in QoL, especially when other problems remain (e.g., lack of social contacts, unemployment, stigmatization) (27). Improvements in global life aspects, leisure activities, living situation and social relations are often associated with better QoL outcomes (28–30). At the level of the services, patient involvement is associated with more feelings of empowerment and satisfaction (31). The interRAI SQoL-MHA instrument includes all these important aspects and the aim of our study is to explore these associations further, using the items of the SQoL–MHA tool in relation to its four domains: relationship, hope, support and activities, as well as the domain relationship with staff. By identifying the significant determinants for each of the SQoL-MHA domains, professional caregivers, together with users can build a better care plan. In addition, this information can help organizations and policy makers design interventions for mental health rehabilitation, considering each domain and its significant factors, to improve perceived QoL. Another aim of our study is to compare these results across settings within the countries involved, showing its potential for benchmarking, as the interRAI SQoL-MHA is an innovative tool which is standardized across settings and was validated for worldwide use in research and practice.

## Materials and Methods

### Data Source

Data for this study were collected in seven countries: Belgium, Finland, Russia, Brazil, Rwanda, Canada, and Hong Kong (China). Trained interviewers assessed participants with the 46-item interRAI Self-Reported Quality of Life Survey for Mental Health and Addictions (interRAI SQoL-MHA) (32). Respondents were at least 18 years old at the time of participation and were receiving mental health services in the community or inpatient mental health care. An additional sample of people from the general population in the community was also assessed in Canada through telephone-based interviews.

### Ethical Approval

Ethical approval was obtained from the Office of Research Ethics (ORE) at the University of Waterloo (ORE#13848, ORE#20863) for the Canadian, Finnish, Russian, and Hong Kong samples; Southlake Regional Health Center Ethics Board (SRHC REB) (#0006-1819) for the Canadian transitional care sample; Ethical Committee Research from Centro Universitário São Lucas Ji-Paraná (CAAE 29517319.9.0000.5297) and Ethical Committee Research from Universidade Luterana do Brasil (CAAE 60213316.9.0000.5349) for the Brazilian sample; Ethical Committee Research of KU Leuven–University of Leuven (Belgium) (S61488) for the Belgian sample and University of Rwanda (No 071/CMHS IRB/2020) for the sample from Rwanda.

### Measures

The interRAI SQoL-MHA consists of 46 items measuring service users' subjective quality of life. The survey was constructed with the purpose of learning what life is like for the user of mental health services and examining how well a program is providing services to this person. Each item is constructed as a 5-point Likert scale based on frequency of the item being true in the person's experience: 0 (never), 1 (rarely), 2 (sometimes), 3 (most of time), and 4 (always). The survey is multi-dimensional and was validated with a Canadian dataset and was later further fine-tuned and validated with an international dataset of 6 countries. The SQoL-MHA instrument was found to have a high reliability, face-validity, and construct-validity across settings and countries (32).

Three additional items to the interRAI SQoL-MHA were used in the analysis: “Work and education opportunities”, “Satisfied with services” and “Worried about making ends meet” (“financial difficulties”). These items are not used in the calculation of the domain scales, but they are assessed as stand-alone items together with the items of the scales. The sample of the Canadian general population was not assessed with items relating to relationship with staff, as they were not receiving mental health care.

### Analysis

The items of the scale were recoded from a 5-point into a 3-point response: 0–1 (never or rarely), 2 (sometimes), and 3–4 (most of time or always), in order to calculate the scores of the interRAI SQoL-MHA domain scales. This method is consistent with Luo et al. (2021), as the scores “never” and “rarely” had a very low frequency. The approach has also been in used in other interRAI QoL surveys for other care settings (33–36). Each domain scale is calculated as a sum of the recoded items: relationship domain (seven items), hope (eight items), support (five items), and activities (three items) and an additional eight-item staff relationship scale. To assess the statistical significance of the difference in the mean scores of the SQoL-MHA domain scales among countries, we performed ANOVA and GLM adjusted Tukey-Kramer correction for unbalanced samples. To explore the associations between the domain scales of the interRAI SQoL-MHA instrument with each other, as well as the associations with the items from the instrument not belonging to the domain scales, logistic models were built. Using the whole pooled sample, all four domain scales of the SQoL-MHA and the scale of relationship with staff were dichotomized for the logistic models, using the scales as dependent variables. Scores below or equal to the 20th percentile (p20) value were recoded as 0. Scores above the p20 value were recoded as 1. This cut-off value was also applied as the benchmark line in the graphs of the comparisons of settings (34). The models for each of the scales controlled for the setting where the services were delivered (community/inpatient), using the general community population as reference, as well as for the country effect. Dummies for each country were created and Finland was chosen as the reference country, as its model of mental health care has more similarities with most countries involved in the study. Multicollinearity tests were performed for all variables in the models, as high correlations among predictor variables may lead to unreliable and unstable estimates of the regression coefficients. All statistical analysis was performed with software SAS version 9.4.

## Results

The sample consisted of a total of 2,474 people from seven countries: 623 (25.2%) users received inpatient care, 1,207 (48.8%) received community mental health services and a Canadian sample from the general population with a total of 644 people (26.0%) living in the community. The samples from Belgium and Canada were both from inpatient and outpatient services. The samples from Brazil, China and Russia came from outpatient care and those from Finland and Rwanda from inpatient facilities. Table 1 shows the distribution of the samples according to gender and age, as well as the percentage of people who had a partner. There was no data from Russia available for these characteristics. The sample from China (outpatient) showed the highest percentage of women (72.7%) and Rwanda inpatient care the lowest (8.2%). The gender distribution of the total sample was of 51% female. The outpatient sample from Canada consisted of people of age 45 or older, contrasting with all other samples, especially with Finland and Rwanda which had the youngest distribution of the population. The outpatient samples from Brazil and China showed the highest percentages of people reporting to have a partner, both about 44%, while the percentage of the total study population was 32.7%.

**Table 1:** Characteristics of the samples of the participating countries and settings.

**Country and setting**	**Female (%)**	**Age (% 45 and older)**	**Client has a partner (%)**	***N***
Belgium inpatient	39.0	60.8	21.2	181
Belgium outpatient	36.4	54.5	24.7	234
Brazil outpatient	58.3	55.3	44.2	570
Canada general community	59.6	63.1	–	644
Canada inpatient	53.9	51.8	–	170
Canada outpatient	51.4	100.0	21.6	148
China outpatient	72.7	61.8	43.6	55
Finland inpatient	44.3	24.1	–	174
Russia outpatient	–	–	–	200
Rwanda inpatient	8.2	18.4	16.3	98
Total population in the study	51.2	56.7	32.7	2474

**Figure 1:**
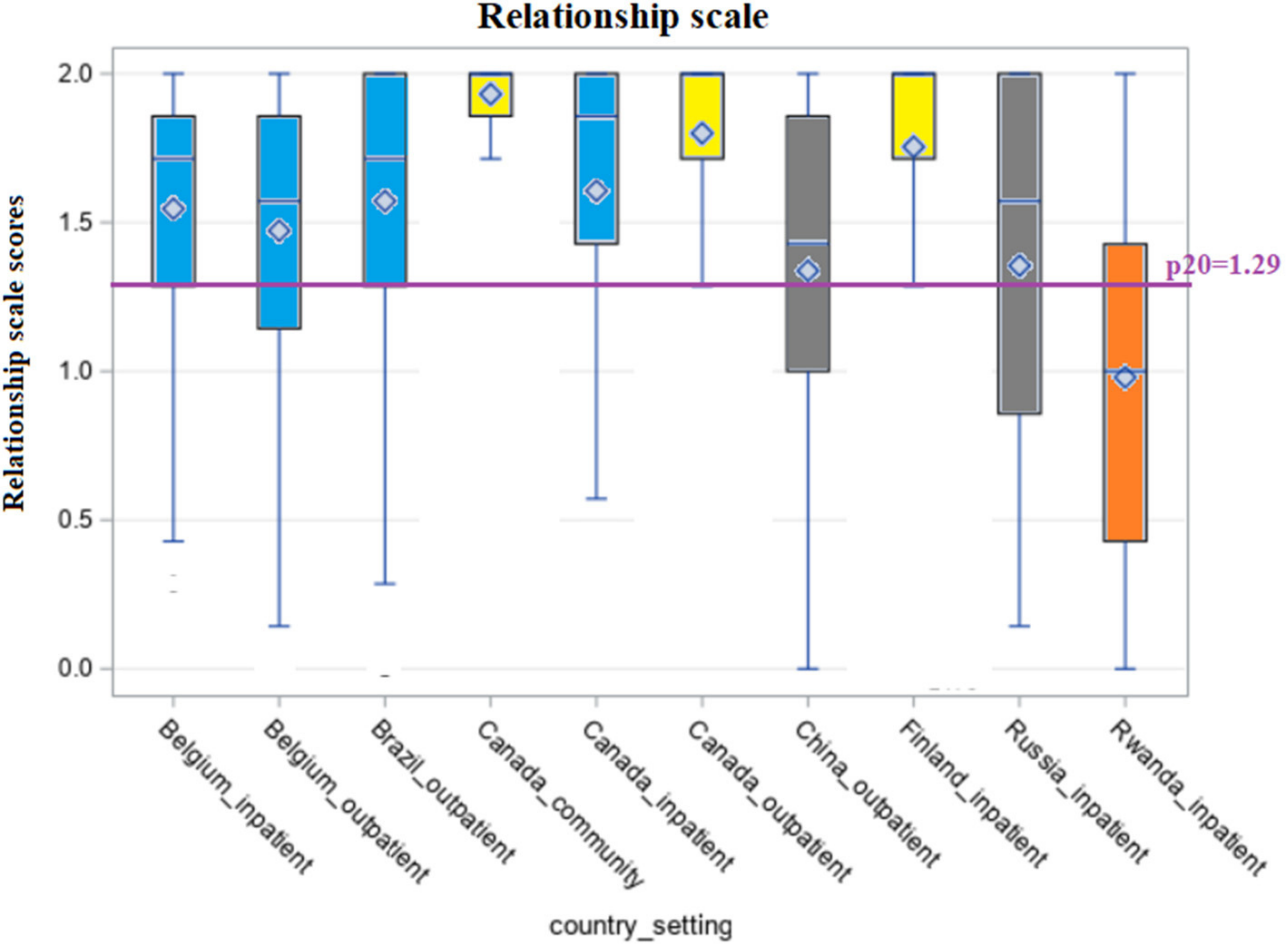
Distribution of scores of the scale “Relationship” across countries and settings.

**Figure 2:**
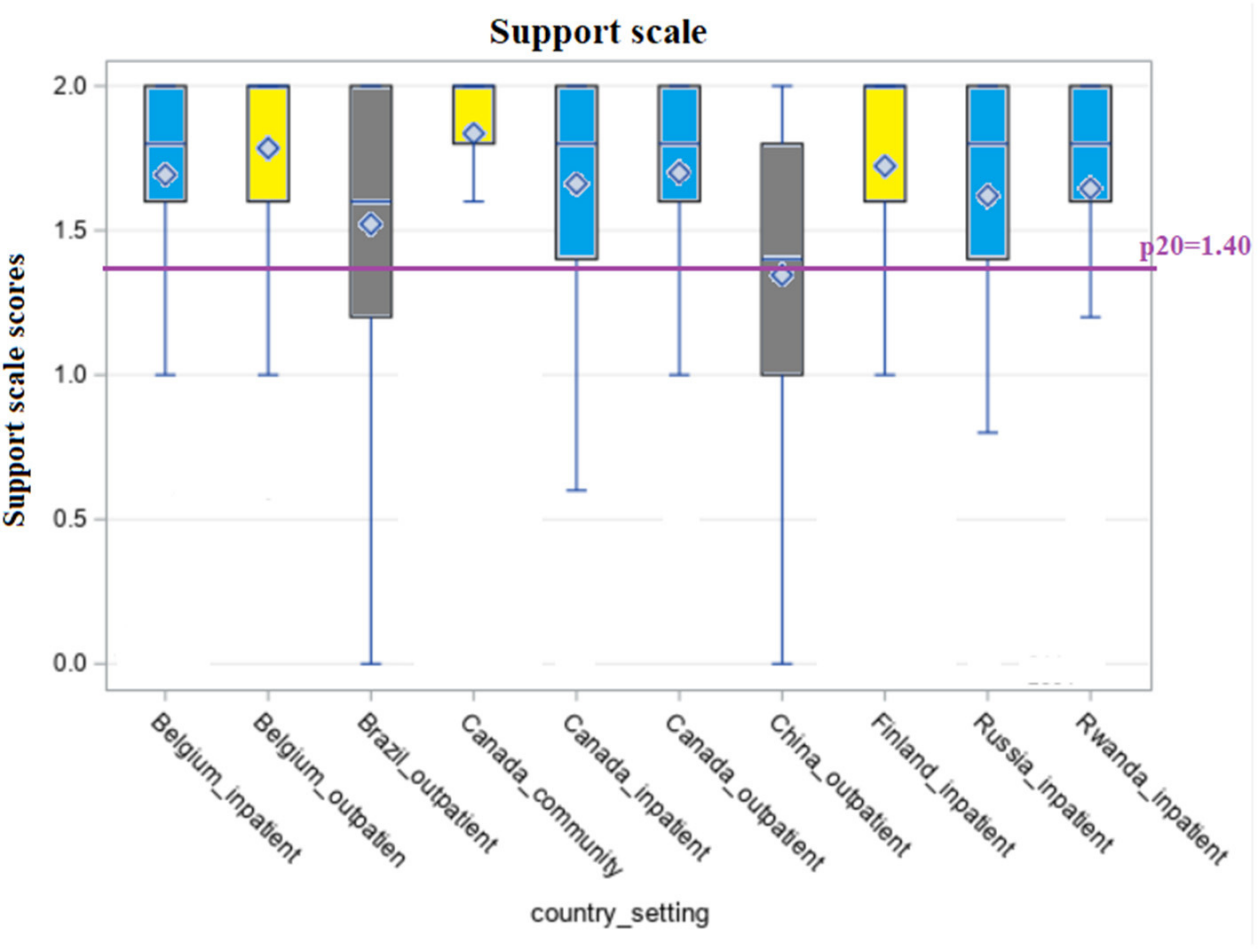
Distribution of scores of the scale “Support” across countries and settings.

**Figure 3:**
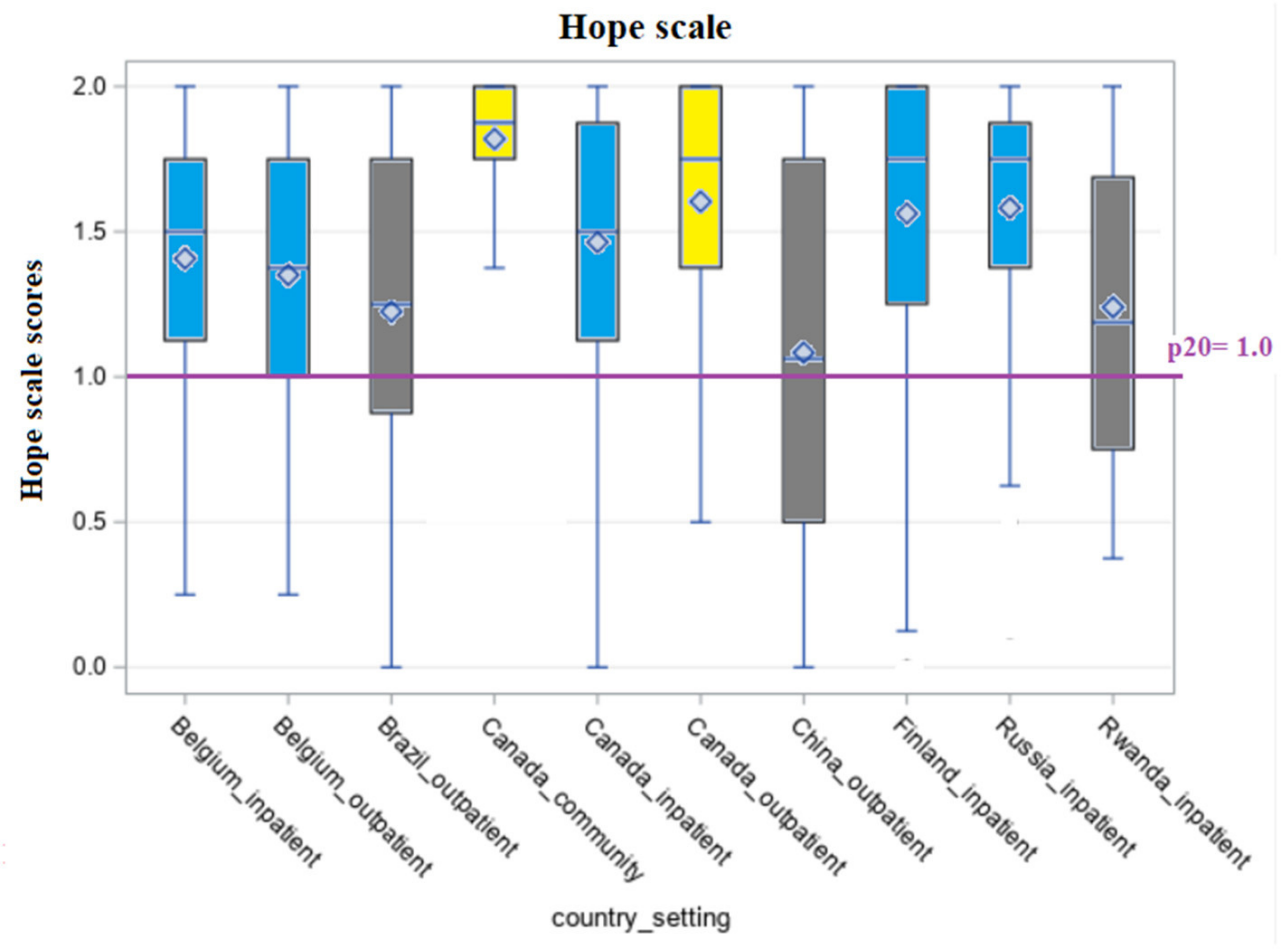
Distribution of scores of the scale “Hope” across countries and settings.

**Figure 4:**
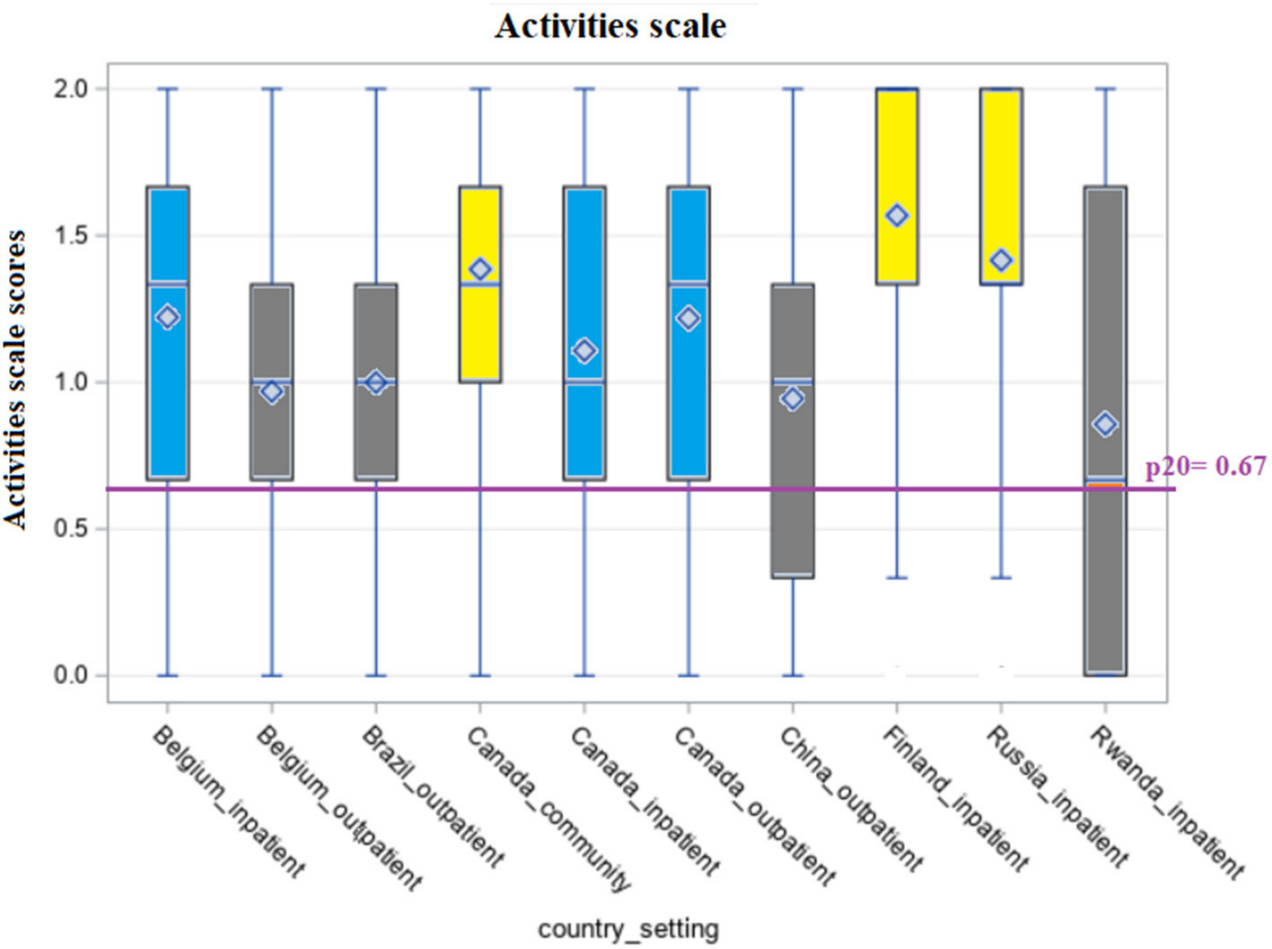
Distribution of scores of the scale “Activities” across countries and settings.

**Figure 5:**
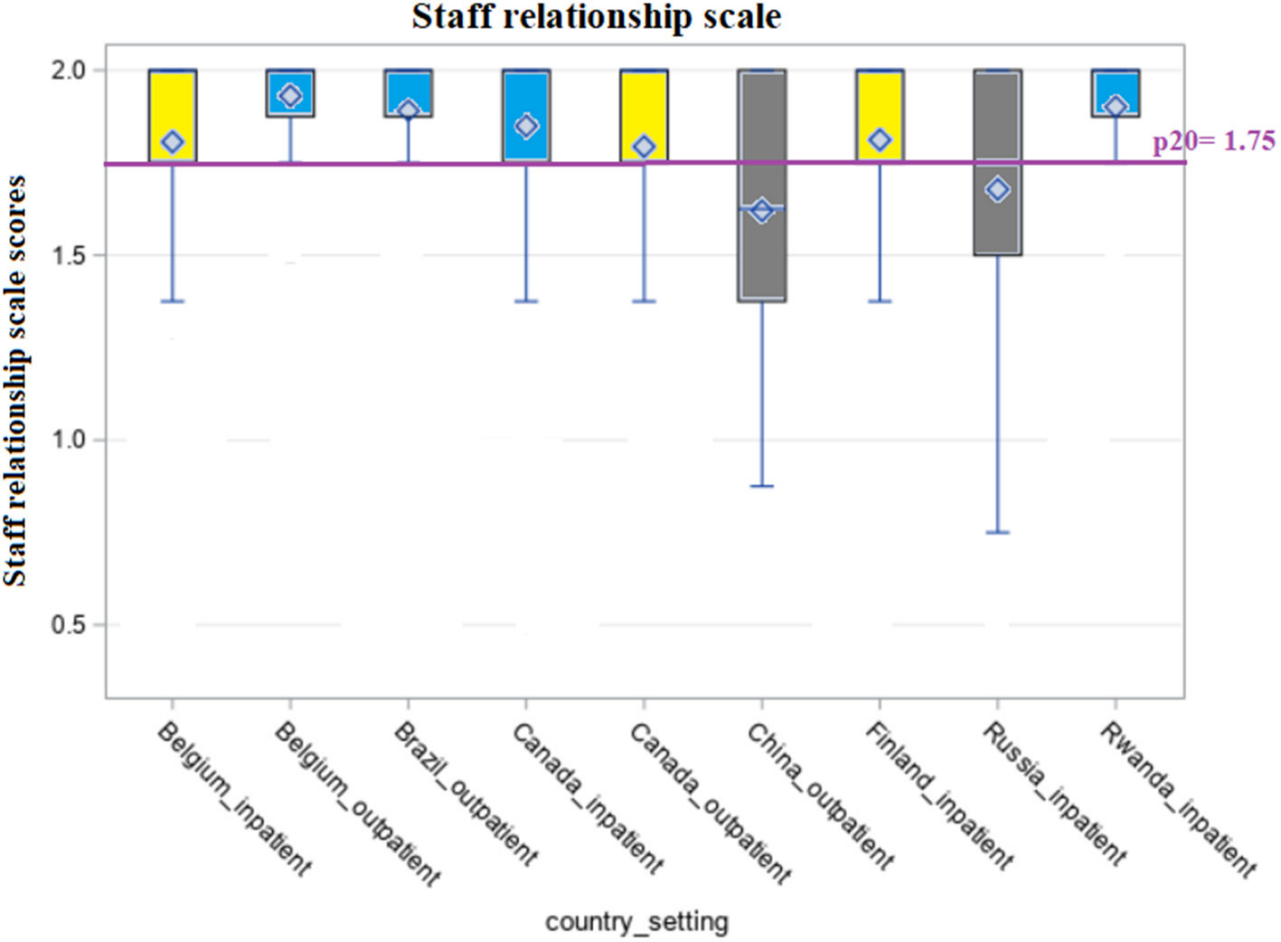
Distribution of scores of the scale “Relationship with staff” across countries and settings.

Figures 1–5 shows the boxplots for the scores of each of the interRAI SQoL-MHA domain scales. Same colors indicate that the samples have means which are not significantly different from each other. Higher scores in the scales mean better scores for each domain scale. As the samples are not representative for each country, the results should be seen as a comparison between samples, and not as comparison between countries. For the relationship scale (Figure 1) we can differentiate four samples with means that are not significantly different from each other (Belgium inpatient and outpatient, Brazil outpatient and Canada inpatient). Another group, with means not significantly differing, consisted of Russia inpatient and China outpatient (respectively 1.35 and 1.34). The sample from the general community population in Canada had the best scores for the relationship scale (mean = 1.91) and the sample from Rwanda the lowest (mean = 0.98), also being the only one with the means below the benchmark of the 20th percentile (p20 = 1.29). Figure 2 shows that only the sample from China outpatient had the mean below the benchmark of 1.4 for the support scale. Figures 3, 4 shows that for the domains hope and activities, none of the samples had means below the p20 line, respectively at the benchmark values of 1.0 and 0.67, although the samples for Rwanda and China had many people with scores below the benchmark lines. In regards to the domain relationship with staff (Figure 5), the samples from Russia inpatient and China outpatient scored lower than the benchmark of 1.75. This is the domain with the highest scores when compared to all other domains and the sample from Rwanda scored high (mean = 1.90), as well as the outpatient samples from Belgium (mean = 1.93) and Brazil (mean = 1.89) and the sample from inpatient care in Canada (mean = 1.85).

Table 2 shows the multivariate logistic models for each of the domains of the interRAI SQoL-MHA instrument. There was no collinearity in the models, as all measures fell within acceptable limits for tolerance and variance inflation factors (limit: VIF <5, most measures were under 2). The analysis of the logistic models did not include missing data. Missing responses on the items of the scales were very limited, mostly between 3 and 5%. The item about work and education opportunities was the only one with more missing values, with a total of 8.5% missing. The domain scales of the SQoL-MHA were in all cases significantly associated with each other in bivariate analysis (see Supplementary Material – Table 1) and in the adjusted logistic models, except for relationship with staff; meaning that each domain scale yielded significant odds ratios for the scores of the other scale. Higher scores in the scale of “support” for example, were associated with higher scores in the domain scale “relationships”, “hope” and “activities”. For the scale of relationship with staff, only the support and relationship domain scales showed a significant association.

**Table 2:** Adjusted logistic models for the interRAI SQoL-MHA domain scales.

	**Relationship**			**Support**			**Hope**			**Activities**			**Relationship with staff**		
**Determinants**	**OR**	**CI−**	**CI+**	**OR**	**CI−**	**CI+**	**OR**	**CI−**	**CI+**	**OR**	**CI−**	**CI+**	**OR**	**CI−**	**CI+**
Support	4.50^***^	3.32	6.12	–	–	–	2.01^***^	1.48	2.73	1.88^***^	1.43	2.48	3.15^***^	2.25	4.41
Hope	3.16^***^	2.3	4.30	1.96^***^	1.45	2.65	–	–	–	3.94^***^	2.98	5.22	1.36	0.96	1.93
Activities	2.55^***^	1.91	3.41	1.93^***^	1.47	2.54	4.17^***^	3.14	5.54	–	–	–	0.98	0.70	1.37
Relationship	–	–	–	4.39^***^	3.22	5.96	3.17^***^	2.32	4.32	2.49^***^	1.87	3.31	2.53^***^	1.81	3.54
Financial difficulties	0.53^**^	0.38	0.73	0.99	0.75	1.34	0.37^***^	0.26	0.53	1.29	0.99	1.67	0.82	0.58	1.15
Work and education	1.17	0.87	1.57	1.32^*^	1.01	1.72	2.70^***^	2.04	3.57	1.34^*^	1.05	1.72	1.20	0.89	1.63
Satisfied with services	1.48	0.69	3.20	9.95^***^	5.74	17.25	1.33	0.62	2.87	1.11	0.62	2.00			
Settings (ref = general community)															
Outpatient	0.11^**^	0.04	0.26	0.86	0.44	1.68	0.21^*^	0.09	0.48	0.29^***^	0.17	0.52	(Ref = outpatient.)		
Inpatient	0.11^***^	0.05	0.24	0.52^**^	0.31	0.85	0.19^**^	0.09	0.39	0.59	0.39	1.00	0.43^**^	0.26	0.72
Countries (ref = Finland)															
Belgium	0.19^**^	0.08	0.50	1.79	0.84	3.82	2.17^*^	1.02	4.61	0.36^*^	0.16	0.81	1.72	0.88	3.37
Brazil	0.67	0.25	1.82	0.38^*^	0.16	0.87	1.52	0.66	3.53	0.56	0.23	1.35	1.85	0.83	4.13
Canada	0.45	0.18	1.16	1.07	0.51	2.26	2.61^*^	1.21	5.65	0.31^**^	0.13	0.69	1.23	0.63	2.41
China	0.38	0.12	1.23	0.34^*^	0.12	0.97	1.08	0.37	3.14	0.68	0.23	1.98	0.21^**^	0.08	0.57
Russia	0.05^***^	0.02	0.13	2.36	0.97	5.73	4.87^**^	1.85	12.84	0.81	0.31	2.12	0.33^**^	0.15	0.71
Rwanda	0.07^***^	0.03	0.18	4.42^**^	1.83	10.67	1.69	0.74	3.90	0.16^***^	0.07	0.39	7.05^***^	2.81	17.66
	*c* = 0.88			*c* = 0.82			*c* = 0.88			*c* = 0.79			*c* = 0.77		

*** *p < 0.0001;*

** *p < 0.01;*

* *p < 0.05*.

*OR, Odds ratios; CI-,Lower confidence interval (95%); CI+, Upper confidence interval (95%); c, c-index for goodness of fit*.

The first model was constructed with the relationship domain scale as the dependent variable. The three other domain scales “support”, “hope” and “activities” were significantly associated with this scale and especially the support scale showed a high odds ratio (OR = 4.50 CI = 3.32–6.12). The item “financial difficulties” had a significant and inverse association with relationship scores (OR = 0.53 CI = 0.38–0.73). The same significant and inverse association was found for the inpatient (OR = 0.11 CI = 0.04–0.26) and outpatient (OR = 0.11 CI = 0.05–0.24) settings when compared with the general community population. Both settings yielded similar odds ratios. Controlling for the countries, Belgium, Russia and Rwanda showed a significant inverse association with the relationship scale, showing more likely to have poorer scores in the relationship scale when compared with Finland.

The second logistic model had the support domain scale as dependent variable. The other domain scales “relationship”, “hope” and “activities” were significantly associated with support. The item “work and education opportunities” showed a positive relationship with the support scale, as well as the item “satisfied with services”, which had a high odds ratio (OR = 9.95 CI = 5.74–17.25). The inpatient setting showed an inverse and significant relationship with the score of the support scale, meaning that the inpatient setting was associated with lower scores for perceived support (OR = 0.52 CI = 0.31–0.85). Controlling for the countries, Brazil and China had a significant association with lower scores on the support scale, when compared with Finland, while Rwanda showed a positive significant association.

The logistic model for the hope domain scale yielded a positive and significant association for all other domain scales “relationship”, “hope” and “activities” and for the item “work and education opportunities” (OR = 2.70 CI = 2.04–3.57). The item “financial difficulties” had a low odds ratio in the model (OR = 0.37 CI = 0.26–0.53), showing that financial problems were associated with lower scores in the hope scale. Controlling for the setting, inpatient and outpatient settings were both associated with lower scores for hope, when compared to the general community population. The countries Belgium, Canada and Russia were significantly associated with higher scores in the hope scale.

The fourth logistic model had the activities domain scale as dependent variable. Positive and significant associations were found for the domain scales “relationship”, “hope” and “activities”, as well as for the item “work and education opportunities” (OR = 1.34 CI = 1.05–1.72). Belgium, Canada and Rwanda were inversely associated with the scores of the activities scale, in comparison with Finland.

The fifth logistic regression model showed the associations for the domain scale “relationship with staff”. Only the domain scales “support and “relationship” were significantly associated with the dependent variable. Controlling for the setting, inpatient care yielded an odds ratio of 0.43 (CI = 0.26–0.72) in comparison with the outpatient setting, representing an inverse association with the score of relationship with staff. China and Russia had low odds ratios, but Rwanda yielded a high odds ratio for the scale of relationship with staff (OR = 7.05 CI = 2.81–17.66).

## Discussion

This cross-country study showed benchmarking comparisons across settings and countries and pointed out some significant associations between items and the domain scales of the self-report interRAI SQoL-MHA tool. The results showed that positive QoL outcomes are achievable in all nations, including low resource nations like Rwanda.

The results from the logistic models showed significant associations between the scales of the SQoL-MHA instrument, as well as significant associations between some items of the instrument and these scales. The item “work and education opportunities” was significantly associated with the domain scales “support”, “hope” and “activities”. This is consistent with scientific literature, as mental health clients who are offered more opportunities to work or to receive an education, feel more empowered and have more feelings of hope and support (37, 38). According to Shepherd, “*employment provides not only an income, but improves social contacts and social support, status and identity, a means of structuring and occupying time and a sense of personal achievement*” (39). In addition, work makes daily life more fulfilling and leisure time more meaningful (40). Among several types of profiles of inpatient and outpatient mental health service users, competitive employment is often viewed by users as an important goal in their rehabilitation path (41–44).

Research from the OECD shows that unemployment rates are generally two times higher for people with a mental disorder compared to individuals without such a disorder (45). Moreover, the presence of a mental illness is associated with higher food insecurity and problems to afford adequate housing, as well as homelessness (46, 47). Our results showed that the perception of financial difficulties (item of the SQoL-MHA “financial trade-offs”) was significantly and inversely associated with the domain scales “relationship” and “hope”. This is consistent with literature as evidence shows that subjective feelings of financial hardship are associated with shame, self-stigma and hopelessness (48–50). In addition, subjective financial hardship tends to be more associated with mental health problems than objective financial hardship, emphasizing the importance of the assessment of subjective financial difficulties (48).

The item “satisfied with services” was significantly associated with the “support” scale. Literature shows associations between frustration with psychiatric services and an inadequate relationship with one's contact person (51). In addition, studies suggest the value of encouraging treatment relationships to develop into positive bonding, so that care users feel supported (52). Research also shows that satisfaction and positive feelings of wellbeing are associated with hope and optimism, as well as greater involvement in society and better relationships. According to Gallagher et al., hope and optimism also contribute positively to the components of social well-being (53). In addition, a high level of self-esteem combined with strong social support, has proven to make individuals less vulnerable to stressors, being associated with mental well-being, happiness, adjustment, success, academic achievements and satisfaction. It is also associated with better recovery after severe diseases (54).

In our study, the inpatient setting showed a significant inverse association for the domain scales “relationship”, “hope”, “support” and the scale “relationship with staff”, when compared to the general community. For the domains “support”, “hope” and “relationship with staff”, the inpatient setting yielded lower scores than the outpatient setting. This means that the inpatient setting is associated with relatively lower (poorer) scores in these quality of life domains. To our knowledge, there is no scientific paper showing comparisons/benchmarking of inpatient and outpatient mental health using self-reported QoL measures. Numerous publications compare the user's characteristics or effectiveness of treatment in both settings (55–59), but they do not focus on self-reported QoL measures. An explanation for a poorer perception of “relationship with staff” in the inpatient setting may lie in the concept of expressed emotion. This means that in the inpatient setting, where users are in contact with staff often on a daily basis, professional caregivers may express more criticism and or hostility, or may express over involvement toward the client. In addition, in institutional settings, negative staff reactions may occur more often, as clients tend to have more difficult behaviors than those in outpatient settings (60). Without adequate training, this can lead to negative symptoms, worse functioning or clients' relapse, as well as professional caregivers' feelings of low personal accomplishment and frustration (61, 62). The scale of “support” showed an inverse association for the samples from Brazil and China and the scale of “relationship with staff” for the samples of Russia and China. The sample of Rwanda, however, showed high odds ratios for both these scales, meaning very high positive association with better scores in support and relationship with staff, when compared to Finland. In addition, the sample from Rwanda scored lower than Finland for the domain scales “relationship”, as well as Belgium and Russia; and for the scale “activities,” and so did Belgium and Canada. These results were also illustrated in the benchmarking graphs. This means that benchmarking of QoL measures is multifaceted, and samples can perform well in some indicators and poorer in others. To our knowledge, this is the first paper to compare inpatient and outpatient care in a cross-country sample. Although the samples are not representative for the countries, they give an indication of the possibilities for benchmarking using the interRAI SQoL-MHA instrument.

The study has some implications worth mentioning. In literature, the lack of studies comparing the QoL of users receiving services in different settings is striking. This can be due to low coordination between settings and the use of many different instruments to assess QoL. The interRAI SQoL-MHA instrument was developed to be used in different settings and, as it has been validated in several countries, can be applied to be used for benchmarking across settings and countries. Moreover, the associations between the scales and items from the SQoL-MHA tool point out the importance of psychosocial rehabilitation in order to reintegrate people with mental health illnesses into the work environment and the community. The item “financial difficulties” and “work and education opportunities” showed significant associations in several QoL domains. In practice, programs like Individual Placement and Support (IPS) proved to be an effective intervention across different settings and economic conditions, leading to competitive employment for people with mental health problems, when compared to traditional vocal rehabilitation. Since it first started in the U.S., it was later also implemented in Europe, Canada, and Australia (63–65).

Our study showed the opportunities offered by the interRAI self-reported Quality of Life instrument (interRAI SQoL-MHA) regarding research and practice, as a validated evidence-based instrument. This tool can be applied in different mental health care settings, in a standardized way, showing possibilities for comparison across countries and settings (benchmarking). Policy makers can view these results as a precedent for coordination across settings, and even countries, within the mental health care framework. Through the use of the same instrument for self-reported QoL, the study showed the possibilities for comparisons and benchmarking. Future research with larger and country representative datasets can provide relevant information to drive policy toward better quality of care and integration across settings. As care users often have a complex care pathway, it is essential to have an effective transfer of information, with the utilization of standard measures. The interRAI instruments offer this standardization, as well as enable evidence-based decision making. By combining the interRAI SQoL-MHA tool with the interRAI-MH or interRAI-CMH instruments, a comprehensive view of subjective quality of care and the objective aspects of care and care needs can be assessed.

## Strengths and Limitations

The main limitation of the study is that the samples are not balanced and cannot be considered representative of the population of the countries involved, so results should not be generalized. However, as noted by Thompson and Forbes (66) estimates of association remain relatively robust even in highly biased samples. A major strength of the study is the application of the interRAI SQoL-MHA in different settings and countries, as the tool is standardized and has been validated to be used in different mental health care settings worldwide.

## Data Availability Statement

The dataset presented in this article are not readily available because the dataset is stored in the secured interRAI server. The dataset can only be available with permission from interRAI. Requests to access the dataset should be directed to prof. John Hirdes, hirdes@uwaterloo.ca.

## Ethics Statement

The studies involving human participants were reviewed and approved by the Office of Research Ethics (ORE) at the University of Waterloo (ORE#13848, ORE#20863) for the Canadian, Finnish, Russian, and Hong Kong samples; Southlake Regional Health Center Ethics Board (SRHC REB) (#0006-1819) for the Canadian transitional care sample; Ethical Committee Research from Centro Universitário São Lucas Ji-Paraná (CAAE 29517319.9.0000.5297) and Ethical Committee Research from Universidade Luterana do Brasil (CAAE 60213316.9.0000.5349) for the Brazilian sample; Ethical Committee Research of KU Leuven–University of Leuven (Belgium) (S61488) for the Belgian sample and University of Rwanda (No 071/CMHS IRB/2020) for the sample from Rwanda. The patients/participants provided their written informed consent to participate in this study.

## Author Contributions

JdA, CV, JHe, HL, JPH, AH, BU, and MS prepared the concept and design of the study. JdA performed the statistical analysis, made the presentation of the results and wrote the manuscript. JdA, CV, JHe, HL, JPH, AH, BU, DG, and MS, discussed the results and made critical revisions to the manuscript. All authors contributed to the article and approved the submitted version.

## Conflict of Interest

The authors declare that the research was conducted in the absence of any commercial or financial relationships that could be construed as a potential conflict of interest.

## Publisher's Note

All claims expressed in this article are solely those of the authors and do not necessarily represent those of their affiliated organizations, or those of the publisher, the editors and the reviewers. Any product that may be evaluated in this article, or claim that may be made by its manufacturer, is not guaranteed or endorsed by the publisher.
