# Supplementary material for: An International Pilot Study of Self-Reported Quality of Life in Outpatient and Inpatient Mental Health Settings
Source: Front Psychiatry. 2021 Aug 5;12:719994. doi: 10.3389/fpsyt.2021.719994 (PMC8374624; doi:10.3389/fpsyt.2021.719994)
Supplement: Supplementary file 1 [file Table_1.docx]

Supplementary material:

Table 1 – Correlation matrix for the variables in the logistic models

|  | Relationship | Hope | Support | Activities | Relationship with staff | Financial difficulties | Work and education | Satisfied with services |
| --- | --- | --- | --- | --- | --- | --- | --- | --- |
| Relationship | - | 0.42*** | 0.36*** | 0.35*** | 0.25*** | -0,13*** | 0.18*** | 0.01 |
| Hope | 0.42*** | - | 0.34*** | 0.41*** | 0.13*** | -0.18*** | 0.32*** | 0.02 |
| Support | 0.36*** | 0.34*** | - | 0.29*** | 0.26*** | -0.11*** | 0.21*** | 0.17*** |
| Activities | 0.35*** | 0.41*** | 0.29*** | - | 0.07** | -0.06** | 0.23*** | 0.01 |
| Relationship with staff | 0.25*** | 0.13*** | 0.26*** | 0.07* | - | -0.05* | 0.07** | 0.19*** |
| Financial difficulties | -0.13*** | -0.18*** | -0,11*** | -0,06** | -0.05* | - | -0.05* | 0.01 |
| Work and education | 0.18*** | 0.32*** | 0.21*** | 0.23*** | 0.07** | -0,05* | - | -0.02 |
| Satisfied with services | 0.01 | 0.02 | 0.17*** | 0.01 | 0.19*** | 0.01 | -0.02 | - |

*** p<0.0001 ** p<0.01 *p<0.05
